# Transglutaminase 2 Has Metabolic and Vascular Regulatory Functions Revealed by In Vivo Activation of Alpha1-Adrenergic Receptor

**DOI:** 10.3390/ijms21113865

**Published:** 2020-05-29

**Authors:** Kinga Lénárt, Attila Pap, Róbert Pórszász, Anna V. Oláh, László Fésüs, András Mádi

**Affiliations:** 1 Department of Biochemistry and Molecular Biology, University of Debrecen, H-4032 Debrecen, Hungary; kinga.lenart@med.unideb.hu (K.L.); papa@med.unideb.hu (A.P.); fesus@med.unideb.hu (L.F.); 2 Doctoral School of Molecular Cell and Immune Biology, University of Debrecen, H-4032 Debrecen, Hungary; 3 Department of Pharmacology and Pharmacotherapy, University of Debrecen, H-4032 Debrecen, Hungary; porszasz.robert@med.unideb.hu; 4 Department of Laboratory Medicine, University of Debrecen, H-4032 Debrecen, Hungary; olaha@med.unideb.hu

**Keywords:** transglutaminase 2 KO, CLAMS, respiratory exchange ratio, heart failure, lactate dehydrogenase, blood pressure, creatine kinase MB

## Abstract

The multifunctional tissue transglutaminase has been demonstrated to act as α1-adrenergic receptor-coupled G protein with GTPase activity in several cell types. To explore further the pathophysiological significance of this function we investigated the in vivo effects of the α1-adrenergic receptor agonist phenylephrine comparing responses in wild type and TG2^-/-^ mice. Injection of phenylephrine, but not a beta3-adrenergic agonist (CL-316,243), resulted in the long-term decline of the respiratory exchange ratio and lower lactate concentration in TG2^-/-^ mice indicating they preferred to utilize fatty acids instead of glucose as fuels. Measurement of tail blood pressure revealed that the vasoconstrictive effect of phenylephrine was milder in TG2^-/-^ mice leading to lower levels of lactate dehydrogenase (LDH) isoenzymes in blood. LDH isoenzyme patterns indicated more damage in lung, liver, kidney, skeletal, and cardiac muscle of wild type mice; the latter was confirmed by a higher level of heart-specific CK-MB. Our data suggest that TG2 as an α1-adrenergic receptor-coupled G protein has important regulatory functions in alpha1-adrenergic receptor-mediated metabolic processes and vascular functions.

## 1. Introduction

Tissue transglutaminase (TG2, EC 2.3.2.13) is a multi-functional protein ubiquitously found in cells [1,2,3]. It possesses both protein cross-linking and guanosine 5′-triphosphate (GTP) hydrolyzing activities [4,5]. In the presence of Ca^2+^, TG2 cannot bind GTP and takes on an open conformation that covalently cross-links proteins producing isopeptide bonds between glutamine and lysine residues through transamidase activity [6,7]. However, in GTP-bound conformation, TG2 takes on a closed conformation and may function as a GTPase [8]. Besides, TG2 can also act as a protein disulfide isomerase [9] and a protein kinase [10,11]. Functions of TG2 have been implicated in various biological processes including regulation of the cytoskeleton, cell adhesion, and cell death as catalytically active or just as an interacting protein partner [1,3,12,13,14]. TG2 can be detected both extracellularly and intracellularly, and it may also localize to the nucleus and the plasma membrane of some cell types [1,2,15,16]. Several reports have linked TG2 functions to different types of diseases [17], like cancer [18,19,20], type 2 diabetes [21], neurodegenerative disorders [22,23,24,25,26], and coeliac disease [27].

In the GTP-bound closed form, TG2 may act as a G protein (Ghα) transmitting signal from α1B-adrenoceptor (α1B-AR) [28,29], oxytocin receptor [30,31], thromboxane A2α receptor [32], and follicle-stimulating hormone receptor [33] only through direct interaction with these receptors [34]. In general, G-protein-coupled receptors (GPCRs) are known to interact with heterotrimeric G proteins; however, TG2/Ghα forms a heterodimer with calreticulin (Ghβ) [30,34] which functions similarly to heterotrimeric G proteins. Receptor activation switches of the TG activity by exchanging GDP to GTP. GTP-bound TG2/Ghα dissociates from Ghβ then directly activates phospholipase Cδ1 (PLCδ1) leading to phosphoinositide hydrolysis and an increase in intracellular Ca^2+^ concentrations [35,36,37]. Moreover, TG2/Ghα can regulate other signaling pathways including inhibition of adenylyl cyclase (AC) activity [38] and direct activation of large-conductance Ca^2+^-activated K^+^ channels’ vascular smooth muscle cells [39]. Signaling by TG2/Ghα is terminated when GTP is hydrolyzed to GDP by its intrinsic GTPase activity and TG2/Ghα associated with Ghβ [34].

Strategies for unfolding the complex biological function of TG2 involve the application of small molecules that can inhibit TG2 activities [3,40,41] and intensive studies on identification and characterization of its substrates and interacting partners [3,42,43]. Additionally, two TG2 knock-out (KO) mouse models have been generated using different approaches, by deleting part of exons 5 and 6, containing the active site, and intron 5 through homologous recombination [44] and by the development of a TG2-loxP knock-in mouse, which allowed inactivation of both alleles after cross-breeding with animals expressing *Cre* recombinase [45]. Considering the multifunctionality of TG2, it is quite surprising to learn that the homozygous deletion of TG2 does not result in a lethal phenotype. The TG2^-/-^ animals are viable and fertile and grow up to normal size and weight with no apparent abnormalities in development and organ functions [44,45]. The most probable explanation for the lack of severe differences is that other transglutaminases in mammalian tissues can compensate for the loss of TG2 [2]. However, detailed investigations of KO models have revealed that TG2 participates in the crosstalk between dying and phagocytic cells to ensure tissue integrity [46,47,48], and it is required for proper differentiation and bacterial killing of neutrophils [49,50]. Ablation of TG2 results in impaired wound healing [51], autoimmunity [52], and hyperglycemia [53]. 

Besides, we have recently shown that TG2^-/-^ mice have decreased cold tolerance compared to TG2^+/+^ due to deficiency in adipocyte browning and low mobilization of fatty acids in gonadal white adipose tissue [54]. The browning mechanism of white adipose tissue involves the activation of heat-producing beige cells through the β-AR signaling pathway [55]. However, β-AR-deficient mice can also increase the thermogenic function of their white adipose tissue in response to mild cold exposure [56] suggesting the existence of other contributing pathways. As we measured high levels of norepinephrine in serum samples of mice after cold exposure [54], we hypothesize that such a mechanism could be the activation of α1-ARs. Cross-talk between α- and β-AR signaling has been proposed in other models [57] in which TG2 as a G protein could play an important role. If so, then activation of α1-AR must reveal physiological differences other than cold tolerance as well between the TG2^+/+^ and TG2^-/-^ strains. Although the G protein function of TG2 has been revealed in several cell types (e.g., heart and smooth muscle cells, fibroblasts, endothelial cells, hepatocytes) [58], its importance has not been investigated extensively in vivo yet. To substantiate the physiological role of TG2/Ghα we injected both TG2^+/+^ and TG2^-/-^ mice intraperitoneally with the specific α1-AR agonist phenylephrine [59] or the specific β-AR agonist CL-316,243 (CL) [60] as control. While TG2^+/+^ and TG2^-/-^ mice responded to CL treatment similarly, TG2^-/-^ mice had significantly lower respiratory exchange ratio (RER) and lower level of tissue damages especially in the heart compared to TG2^+/+^ animals after phenylephrine treatment. Furthermore, phenylephrine treatment has no obvious effect on blood pressure in TG2^+/+^ mice in contrast to the TG2^-/-^ ones. On the whole, our data support the role of TG2/Ghα in maintaining regulation and responsiveness of the circulatory system.

## 2. Results

### 2.1. Alpha1 But Not Beta3 AR Agonist Treatment Leads to Decreased Physical Activity in Both TG2^+/+^ and TG2^-/-^ Mice

TG2 in some cell types can act as a G protein coupled to α1B-ARs or α1D-ARs; thus, we studied the effect of the α1-AR agonist phenylephrine treatment on both TG2^+/+^ and TG2^-/-^ mice. We also used the β3-AR agonist CL treatment for a comparison of AR signalization pathways in the investigated mice. First, we applied the Comprehensive Lab Animal Monitoring System (CLAMS), which is a system of metabolic cages allowing for simultaneous measurement of several metabolic parameters like total food and water intake, X plane cage movements (XTOT), heat production, O_2_ consumption (VO_2_), CO_2_ production (VCO_2_), and respiratory exchange ratio (RER) [61]. 

**Figure 1:**
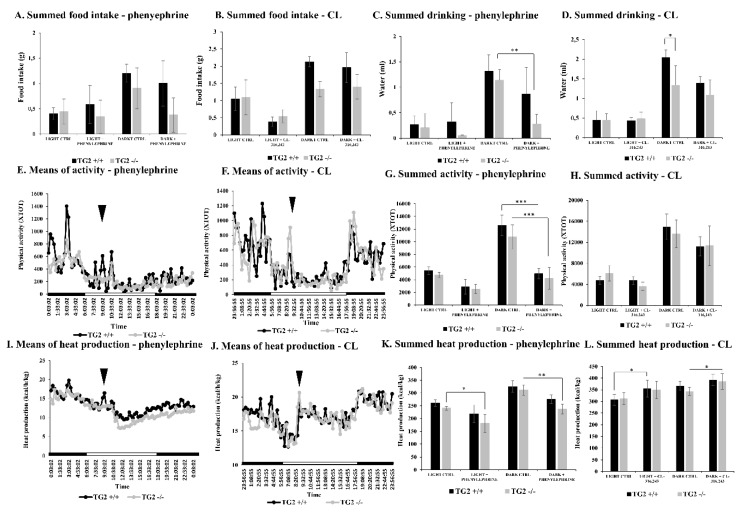
Alpha1 but not beta3 adrenoreceptor (AR) agonist treatment leads to decreased physical activity in both TG2^+/+^ and TG2^-/-^ mice. Total (**A**,**B**) food intake and (**C**,**D**) water consumption are shown before (CTRL) and after (**A**,**C**) phenylephrine or (**B**,**D**) CL-316,243 (CL) treatment for both in the light (9:03 a.m.–6:00 p.m.) and the dark (6:01 p.m.–6:00 a.m.) periods. Mean values of physical activity are presented before and after (**E**) phenylephrine and (**F**) CL treatments. Arrowheads show the time point (9:00 a.m.) when the compounds were injected intraperitoneally (60 nM/g body weight). Day (□) and night (■) periods are indicated on the “X” axes (*n* = 6). Total physical activity is also presented for (**G**) phenylephrine and (**H**) CL investigations before (CTRL) and after the treatments both in the light and dark periods. (**I**,**J**) Mean values of heat generation and (**K**,**L**) total heat generation are shown for (**I**,**K**) phenylephrine and (**J**,**L**) CL experiments. Columns represent the mean values ± SD (*n* = 6). Statistical analyses were carried out using GraphPad Prism 7.0 version, by two-way ANOVA (Tukey’s multiple comparison test; * *p* < 0.05, ** *p* < 0.01 and *** *p* < 0.001).

There was no significant difference in the food intake between the strains throughout all the periods of the experiment (Figure 1A,B). Interestingly, TG2^-/-^ mice drank less water in the dark period of the measurement day compared to the control day; however, it is important to note that when their values were compared to the corresponding TG2^+/+^ values, significant differences were not detected (Figure 1C). Besides this, TG2^-/-^ mice drank less water in the dark period on the control day of the CL-experiment (Figure 1D) that was not observed in the case of the phenylephrine experiment; however, it was physiologically enough for them as it had no significant effect on the investigated physiological parameters.

The physical activity of the strains was not different on the control day either. Intraperitoneal injection of phenylephrine had an effect on TG2^+/+^ and TG2^-/-^ mice similarly (Figure 1E) as it caused a serious decrease in the physical activity of both strains, it was more prominent on the measurement day only in the dark period, which is normally the active time of mice (Figure 1G). However, interestingly, CL treatment did not affect the activity of mice (Figure 1F,H).

The strains generated the same amount of heat on the control day (Figure 1I–L); however, phenylephrine treatment decreased the summed total heat production only in the TG2^-/-^ strain on the measurement day compared to the control day (light period: 181+/−35 kcal/kg vs. 241+/−7.5 kcal/kg, *p* < 0.05; dark period: 238+/19 kcal/kg vs. 312+/−20 kcal/kg, *p* < 0.05; Figure 1I,K). Furthermore, after injection of CL, the TG2^+/+^ mice increased heat production in the light period and the TG2^-/-^ animals in the dark period on the measurement day, but it is more important that there were no differences when the strains were compared to each other in the investigated periods (Figure 1K,L).

### 2.2. Alpha1 But Not Beta3-AR Agonist Treatment Results in a Decrease of the Respiratory Exchange Ratio in TG2^-/-^ Mice Compared to TG2^+/+^ Animals

**Figure 2:**
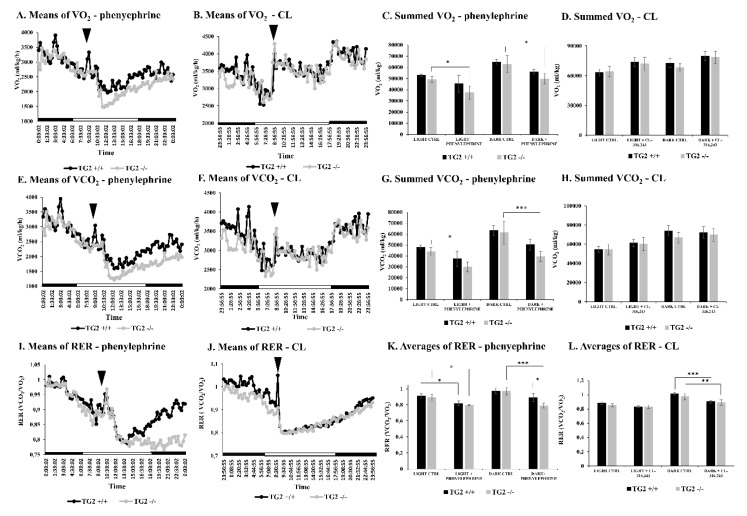
Treatment with the alpha1-AR agonist leads to a significant decrease in the respiratory exchange ratio (RER) of TG2^-/-^ mice compared to TG2^+/+^ animals. Mean values of (**A**,**B**) O_2_ consumption (VO_2_), (**E**,**F**) CO_2_ production (VCO_2_), and (**I**,**J**) average values of the respiratory exchange ratio (RER) are presented before and after the treatment with (**A**,**E**,**I**) phenylephrine or (**B**,**F**,**J**) CL over the time (*n* = 6). Arrowheads show the time point (9:00 a.m.) when the injection was carried out (60 nM/g body weight for both compounds). Day (□) and night (■) periods are indicated on the “X” axes. Summed values of (**C**,**D**) VO_2_, (**G**,**H**) VCO_2_ and the average values of (**K**,**L**) RER are shown before (CTRL) and after the corresponding treatment for both in the light (9:03 a.m.–6:00 p.m.) and the dark (6:01 p.m.–6:00 a.m.) periods. Columns represent the mean values ± SD (*n* = 6). Statistical analyses were carried out using GraphPad Prism 7.0 version, by two-way ANOVA (Tukey’s multiple comparison test; * *p* < 0.05, ** *p* < 0.01 and *** *p* < 0.001).

VO_2_ values of the strains were the same both in the light and dark periods of the control day without any treatments (Figure 2A–D). Phenylephrine induced a decrease in VO_2_ levels after absorption in the case of both strains. Nonetheless, TG2^+/+^ animals set back the normal value within the light period after the treatment and recovered, but the VO_2_ of TG2^-/-^ mice remained lower (Figure 2A). In contrast, CL treatment evoked a little increase of VO_2_ of the strains, however, in a very similar way when they were compared to each other (Figure 2B). We calculated the total VO_2_ for the light and dark periods before and after the phenylephrine treatment and we found that the values of TG2^+/+^ mice remained unchanged. Although the VO_2_ values of TG^2-/-^ animals were lower after the treatment both in the light and dark periods correlated to their corresponding control periods, there were no significant differences when we compared the values of TG2^-/-^ mice to TG2^+/+^ ones (Figure 2C). Meanwhile, CL treatment did not cause any significant changes in the total VO_2_ values of the strains (Figure 2D). 

Similarly to VO_2_, the VCO_2_ values of the strains were the same both in the light and dark control periods (Figure 2E–H). Phenylephrine treatment caused a decline in VCO_2_ levels of the strains equally, and then they started to increase gradually to the normal levels. However, the value of TG2^-/-^ mice remained a little lower after the treatment (Figure 2E). Interestingly, the CL treatment did not affect the VCO_2_ values of the strains, which remained the same (Figure 2F). We also calculated the total VCO_2_ for the light and dark periods before and after phenylephrine treatment and found that the values of TG2^+/+^ mice remained unchanged. Though similarly to VO_2_, the VCO_2_ values of TG2^-/-^ animals were lower both in the light and dark periods after phenylephrine treatment compared to their analogous control periods, there were no significant differences when we compared them to the values of TG2^+/+^ mice (Figure 2G). Like in the case of VO_2_, the CL treatment did not cause any significant changes in total VCO_2_ values of the strains (Figure 2H). 

RER values (VCO_2_/VO_2_) of TG2^-/-^ and TG2^+/+^ mice were very similar both in the light and the dark periods of the control day before phenylephrine treatment (Figure 2I–K). After absorption of intraperitoneally injected 60 nM/g body weight phenylephrine the RER values of both strains significantly decreased similarly in the first part of the light period on the treatment day demonstrating that the treatment affected. However, while the RER value of the TG2^+/+^ strain started to increase constantly in the second part of the light period, and in the following dark period it reached the level detected before the treatment, RER remained still low in the case of TG2^-/-^ mice (Figure 2I). This resulted in significantly lower RER value of TG2^-/-^ animals compared to TG2^+/+^ ones in the dark period on the measurement day (TG2^-/-^: 0.79+/−0.03 vs. TG2^+/+^: 0.89+/−0.05, *p* < 0.05; Figure 2K). After absorption of intraperitoneally injected 60 nM/g body weight CL in the first part of the light period on the treatment day, the RER values of both strains promptly dropped down demonstrating the physiological effect of CL and then started to increase equally in the second part of the light period (Figure 2J). Consequently, while the RER values of both strains were significantly lower in the dark period on the measurement day compared to the control dark period, there was no difference between the strains (Figure 2L).

### 2.3. Alpha1-AR Agonist Treatment Results in Lower Blood Lactate Levels in TG2^-/-^ Mice

**Figure 3:**
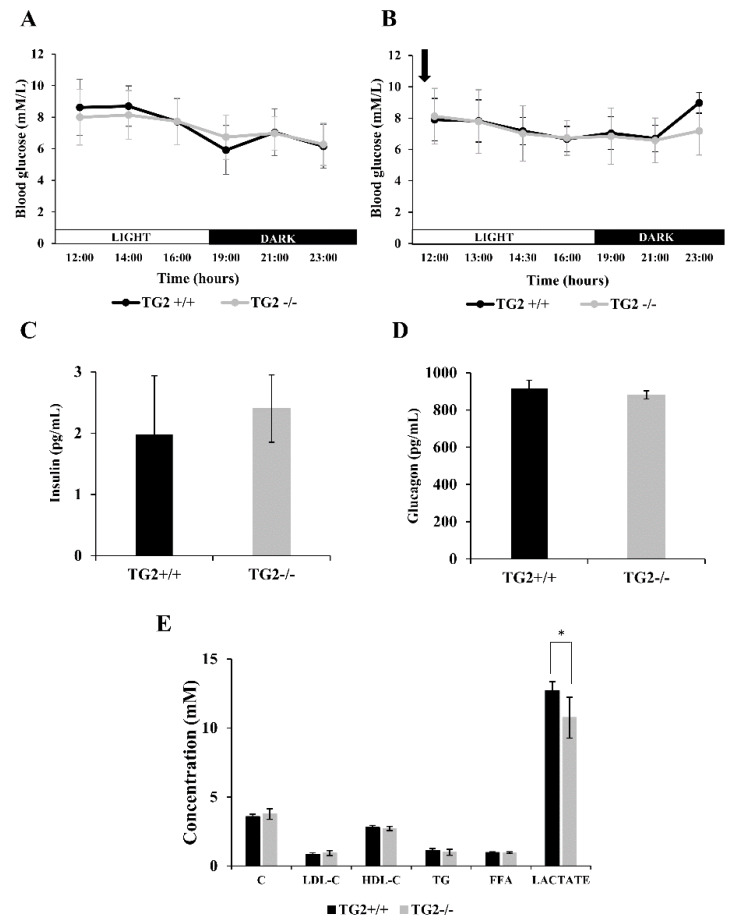
Blood metabolic parameters of TG2^+/+^ and TG2^-/-^ mice after phenylephrine treatment. (**A**) Blood glucose levels of TG2+/+ and TG2-/- mice on the control day. (**B**) Blood glucose levels of TG2^+/+^ and TG2^-/-^ mice after phenylephrine treatment. Day (□) and night (■) periods are shown on the “X” axes. Arrowhead indicates the time point of phenylephrine injection. The experiments were carried out according to the scheme presented in Figure 4A. (**C**) Insulin concentration in serum samples of TG2^+/+^ and TG2^-/-^ mice 13 h after phenylephrine treatment. (**D**) Glucagon concentration in serum samples of TG2^+/+^ and TG2^-/-^ mice 13 h after phenylephrine treatment. (**E**) Serum lipid and lactate concentration values of mice 13 h after phenylephrine treatment. C: cholesterol, LDL-C: low-density lipoprotein cholesterol, HDL-C: high-density lipoprotein cholesterol, TG: triacylglycerol, FFA: free fatty acid. Columns or measurement points represent the mean values ± SD. Statistical analyses were performed using Student’s *t*-test (*n* = 5, *p < 0.05).

Blood glucose levels of TG2^+/+^ and mice TG2^-/-^ were the same on the control day both in the light and the dark periods (Figure 3A), and phenylephrine injection did not lead to changes (Figure 3B). Both insulin and glucagon levels were found similar in the serum samples of the strains obtained at the same time point after the treatment (Figure 3C,D). Furthermore, serum concentrations of lipid fractions cholesterol (C), low-density lipoprotein cholesterol (LDL-C), high-density lipoprotein cholesterol (HDL-C), triglyceride (TG), and free fatty acid (FFA) did not differ after phenylephrine treatment either in TG2^+/+^ or TG2^-/-^ mice (Figure 3E). Interestingly, however, lactate concentration was significantly higher in the serum of TG2^+/+^ mice compared to TG2^-/-^ ones (12.7+/−0.64 mM vs. 10.74+/−1.4 mM, *p* < 0.05) 13 h after the phenylephrine treatment (Figure 3E).

### 2.4. Alpha1-AR Agonist Has a Lowering Effect on Tail Blood Pressure of TG2^+/+^ But Not TG2^-/-^ Mice

**Figure 4:**
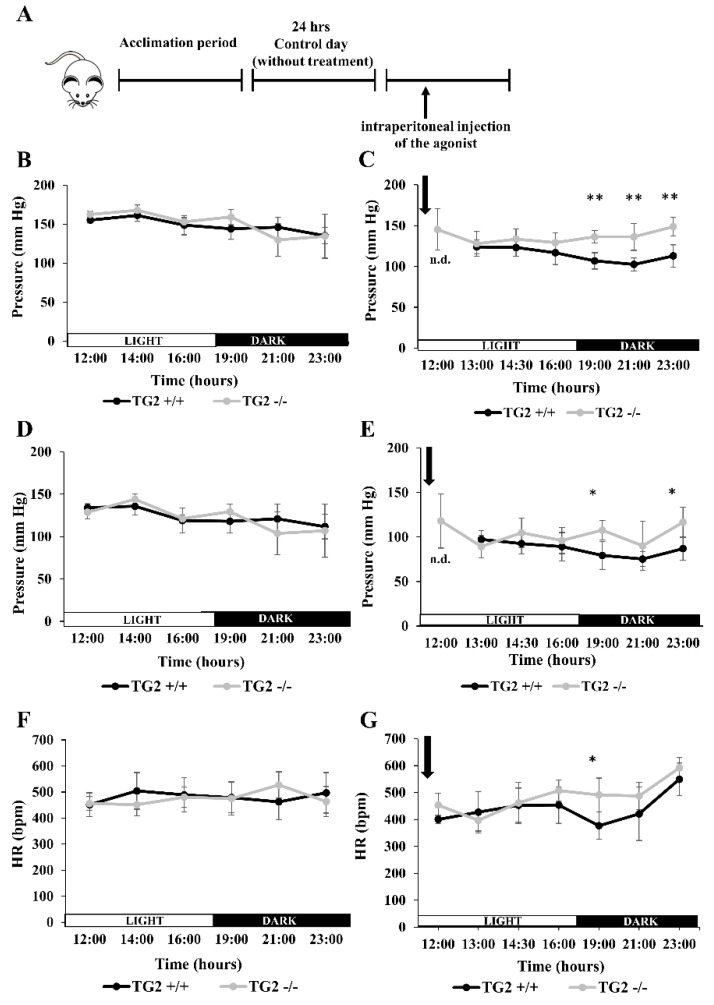
Phenylephrine treatment does not decrease tail blood pressure of TG2^-/-^ mice in contrast to TG2^+/+^. (**A**) Schematic presentation of the experiments. Tail blood pressure (TBP) and heart rate (HR) were measured at the same time points of both the acclimation day and the control day. On the third measurement day, mice were injected intraperitoneally with 60 nM/g body weight phenylephrine at 11:30 a.m. Systolic pressure on the control day (**B**) and the measurement day (**C**); and diastolic pressure on the control day (**D**) and on the measurement day (**E**) are presented separately. (**F**) HR values on the control day (**G**) HR values on the measurement day. Day (□) and night (■) periods are shown on the “X” axes. Arrowheads indicate the time point of phenylephrine injection. Measurement points represent the mean values ± SD. Statistical analyses were carried out using Student’s *t*-test (*n* = 5, * *p* < 0.05, ** *p* < 0.01).

Comparing TG2^+/+^ and TG2^-/-^ mice, we found that both their tail blood pressure (TBP) and heart rate (HR) values were very similar before the treatment (Figure 4B,D,F). Oddly, at the first measurement time point of the experiment, that was 30 min after the injection of phenylephrine, we could not record both systolic and diastolic pressure of all the investigated TG2^+/+^ mice in contrast to TG2^-/-^ animals in which detection of TBP was successful. Besides, while TBP values of the strains were similar in the light period, systolic pressure values and two of the three detected diastolic pressure values were significantly lower in TG2^+/+^ animals compared to TG2^-/-^ mice in the dark period (Figure 4C,E). As a result, TBP values of TG2^-/-^ mice remained unchanged after the phenylephrine treatment, while those of TG2^+/+^ animals were lower in the dark period compared to the control day (Figure 4C vs. Figure 4B; Figure 4E vs. Figure 4D). Furthermore, we did not detect differences in HR values of the strains except the second measurement of the dark period after the treatment, when TG2^+/+^ showed lower levels (Figure 4F,G). 

### 2.5. Alpha1-AR Agonist Evokes a Higher Level of Tissue Damages Monitored by Lactate Dehydrogenase (LDH) Release in TG2^+/+^ than in TG2^-/-^ Mice

**Figure 5:**
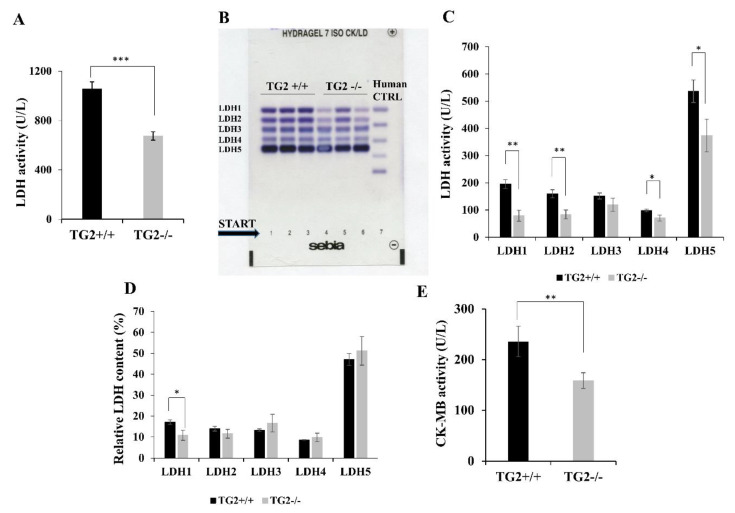
Phenylephrine treatment results in a lower degree of tissue damages reflected in changes of LDH isoenzyme patterns in TG2-/- mice compared to TG2+/+. (**A**) Total lactate dehydrogenase isoenzyme (LDH) enzyme activity values in serum samples of mice 13 h after the phenylephrine treatment. (**B**) Agarose gel electrophoretic separation of serum lactate dehydrogenase isoenzymes (LDH-1 to LDH-5) of TG2^+/+^ and TG2^-/-^ mice (lanes: 1–3 TG2^+/+^, lanes: 4–6 TG2^-/-^, lane 7: human control serum). (**C**) Enzyme activity levels of LDH isoenzymes in serum samples of mice 13 h after the phenylephrine treatment. (**D**) Relative LDH isoenzyme activity values in serum samples of mice 13 h after the phenylephrine treatment. (**E**) CK-MB activity levels in serum samples of mice 13 h after the phenylephrine treatment. Columns represent the mean values ± SD. Statistical analysis was performed using Student’s *t*-test (*n* = 3, * *p* < 0.05, ** *p* < 0.01, *** *p* < 0.001).

Serum samples of TG2^+/+^ mice isolated 13 h after the phenylephrine treatment contained a significantly higher level of LDH compared to TG2^-/-^ animals’ (725+/−34 U/L vs. 1142+/−58 U/L, *p* < 0.001; Figure 5A). Electrophoretic separation and densitometry revealed that four out of the five isoenzymes were present in larger quantities in TG2^+/+^ samples than in TG2^-/-^ ones, namely LDH1, LDH2, LDH4 and LDH5 (Figure 5B,C). The KO/wild type (WT) ratios of the isoenzymes LDH2, LDH4, and LDH5 were similar. However, the KO/WT ratio of LDH1, which may represent a cardiac-specific component, was lower (11%+/−2% vs. 17%+/−1%, *p* < 0.05; Figure 5D). Moreover, the level of CK-MB was also higher in the serum samples of TG2^+/+^ mice compared to TG2^-/-^ animals’ (235+/−30 U/L vs. 158+/−16 U/L, *p* < 0.01; Figure 5E).

## 3. Discussion

The deletion of the multifunctional TG2 does not cause severe phenotype in mice. It is generally explained by the fact that other members of the TG family can be expressed in cells and they may substitute most of the lacking functions of TG2. However, the other mammalian TG do not bind GDP/GTP, consequently, they cannot cover the G protein function of TG2. Nonetheless, other classical G proteins are likely present in TG2-expressing cells, which can assume the role of TG2 as a G protein. Therefore, more severe alterations have been expected in viable TG2^-/-^ mice only under certain stresses and pathological conditions [2].

Phenylephrine is a sympathomimetic amine with epinephrine-related chemical structure selectively binds to α1-ARs and activates them [62]. The G protein-coupled ARs have a 7-transmembrane domain structure, and in general, they can mediate contraction and hypertrophic growth of smooth muscle cells. ARs have three subtypes, which share approximately 75% homology: α1A, α1B, and α1D. Phenylephrine appears to act similarly on all three receptor subtypes [63]. It is well-recognized that TG2 may act as a Ghα protein binding to α1B-ARs or α1D-ARs, but not α1A-ARs [64], independently from its transamidation activity. Therefore, we investigated the effect of phenylephrine treatment on TG2^+/+^ and TG2^-/-^ mice and used CL β3-AR agonist for comparison.

Comparing TG2^+/+^ and TG2^-/-^ mice we found striking differences in RER (ratio of CO_2_ production and O_2_ consumption) values especially in the dark period, several hours after the phenylephrine treatment. In the case of TG2^+/+^ animals, RER dropped, then started to increase gradually from about 0.8 to the level close to 1, which was detected as a control value before injection in both strains. Meanwhile, RER of TG2^-/-^ mice did not start to increase, after dropping and remained low even in the dark period after the treatment. The dark period is normally the active phase of mice, but the development of this deviation is unrelated to this as there was no difference in the physical activity of the animals, which decreased and remained significantly lower for both strains even in the dark period after the injection. It is generally accepted that RER indicates which type of fuel is being preferably metabolized. It ranges from 1.0 where pure glucose is oxidized to 0.7 where pure FFA is oxidized [65]. Accordingly, TG2^+/+^ and TG2^-/-^ mice gained energy mainly from the degradation of carbohydrates before the phenylephrine injection, but after the treatment, they immediately started to utilize FFAs in our experiments. Interestingly, however, TG2^-/-^ animals continued to break down FFAs for a much longer time while TG2^+/+^ mice started to utilize carbohydrates increasingly. The observed approximately 0.1 difference in the RER values after the phenylephrine treatment on a maximum 0.3 scale (from 0.7 to 1.0) reflects a quite different metabolism of the whole organism kept on the same diet and is a surprising finding worth investigating further. It is important to note that activation of both α1A- and α1B-ARs can increase whole-body fatty acid oxidation resulting in a decrease of RER [66]. TG2^-/-^ animals reached the RER values of TG2^+/+^ mice later at the end of the recovery stage, at about 30 h after the treatment (data not shown). These results indicate that TG2 functioning as a Ghα protein in adrenergic receptor signaling may play a role in determining the balance between carbohydrate and fatty acid oxidation. 

We applied CL, a specific β3-AR agonist as a control for the α1-AR agonist phenylephrine. This receptor is located mainly in adipose tissue where it can enhance lipolysis [67]. Consequently, the injection of CL resulted in the prompt decrease of RER values in both TG2^-/-^ and TG2^+/+^ mice, which started to increase gradually in a similar way during the dark period. Other metabolic parameters of the strains were also very similar which shows that TG2 has no role in signal transduction regulated by β3-AR. Β3-AR is also involved in the regulation of thermogenesis in skeletal muscle [68] and fat tissues [69]; but, we did not detect differences in heat production of the strains after β3-AR stimulation. However, the generation of heat decreased in TG2^-/-^ mice and was lower after phenylephrine injection both in the light and the dark periods. We have reported that TG2^-/-^ mice have decreased tolerance to cold exposure [54], and this result may suggest that TG2 potentially affects heat production as an α1B-AR-coupled G protein in mice.

As α1-ARs may have roles in the regulation of various metabolic processes [66,70,71], first we measured blood glucose levels of strains and found them to be similar and normal [72,73] both before and after the phenylephrine injection. Phenylephrine increased glucagon levels compared to the normal values through an unknown subtype of α1-AR as it is described [73], but its levels in the plasma of TG2^-/-^ and TG2^+/+^ animals were similar 13 h after the phenylephrine treatment when the difference in RER values was prominent. These results together with the obtained data of lipid fractions suggest that the most important fuels for cellular metabolism were available in excess for mice fed ad libitum, and their different utilization could result in the dissimilar RER values as discussed above. Phenylephrine is thought to act quickly, within some minutes after absorption, and its estimated half-life is about 2.1–3.4 h [74] as it is extensively metabolized by monoamine oxidase [75]. Therefore, the observed difference in RER values probably was not caused by the presence of phenylephrine directly, but rather long-term effects induced by this agonist.

It has been reported that α1B-AR KO mice had normal blood glucose and insulin levels, but elevated leptin concentrations in the fed state [71]. Leptin is known to stimulate glucose uptake and beta-oxidation by activating AMP-activated protein kinase (AMPK) especially in the skeletal muscle [76]. The activation of AMPK can be mediated by two different mechanisms: firstly, by the direct effect of leptin, or secondly, by its indirect effect through the hypothalamic sympathetic nervous system and α1B-AR mechanism [71,77]. The activation of AMPK by leptin leads to phosphorylation and inhibition of acetyl-CoA carboxylase (ACC) and results in the stimulation of fatty acid oxidation by disinhibition of carnitine palmitoyltransferase 1 (CPT1) in muscle [76]. If TG2 can act as Ghα, then signal transduction of α1B-AR can be deficient at least in part in TG2^-/-^ mice resulting in a higher level of leptin and lower RER, as we observed, compared to TG2^+/+^ after phenylephrine treatment. Higher lactate levels in the plasma of TG2^+/+^ mice probably indicated that they utilized more glucose than TG2^-/-^ ones. 

Phenylephrine is a potent vasoconstrictor that increases cardiac preload without any important direct effect on cardiac myocytes. It can increase blood pressure keeping a slow heart rate through stimulation of vascular baroreceptors. Thus, phenylephrine is generally administered for patients with normal cardiac function but with clinically significant hypotension caused by vasodilation [78]. Phenylephrine can cause pronounced vascular adverse effects, including increases in both systolic and diastolic BP [79], therefore we measured TBP of the mice. We found that both systolic and diastolic BP of TG2^-/-^ animals were similar to TG2^+/+^ values on the control day before the treatment. It is important to note that phenylephrine is known to cause contraction of tail caudal arteries making impossible the detection of TBP until the compound is degraded [80]. Indeed, we could not detect TBP of TG2^+/+^ mice at the first time point 30 min after the phenylephrine injection. However, TBP could be measured in all investigated TG2^-/-^ animals, and their TBP values were comparable with those found at the control day suggesting absence or just low level of contraction in their caudal arteries at the first time point of the measurement in the absence of TG2. Decreased BP response in α1B-AR KO mice, similar to our finding in TG2^-/-^ mice, was demonstrated suggesting that this type of AR is an important mediator of BP [81]. Consequently, if TG2 can act as an α1B-AR-coupled Ghα, then this function could lead to increased smooth muscle cell tone [29,82,83,84] and explain why we did not observe vasoconstriction in the tail of TG2^-/-^ mice. Furthermore, it was shown that TG2 modulated vascular function through its crosslinking-independent manner as well, and TG2^-/-^ aorta was found to be stiffer compared with TG2^+/+^ [85]. Interestingly, the long-term effect of phenylephrine was also apparent in our study, as systolic BP of TG2^+/+^ animals remained significantly lower in the dark period, and their diastolic BP was also lower at two measurement points out of the three in this period. 

Because of its vasoconstrictive effect, phenylephrine can cause severe necrosis in tissues [78]. LDH is a soluble cytoplasmic enzyme that is present in almost all cells and released into extracellular space when the plasma membrane is damaged during necrosis [86]. Quantifying the LDH activity in the serum samples indicates the degree of tissue damages [87], therefore, we measured the LDH activity in serum samples of the strains to compare the level of necrosis caused by phenylephrine treatment. Total LDH activity was pathologically high in both strains compared to normal values 13 h after the injection [88], but it was significantly higher in TG2^+/+^ samples compared to TG2^-/-^ sera. The tetrameric LDH is composed of two types of subunits, the LDH-M, and the LDH-H proteins. These two subunits can form five possible isoenzymes (LDH1-5) that are enzymatically similar but show different tissue distribution. Consequently, the pathological appearance of different isoenzymes in serum can indicate in which tissue or organ the damage occurred [89]. Electrophoretic separation and quantitation of LDH isoenzymes revealed that phenylephrine treatment caused damages in several organs, but these were higher in TG2^+/+^ mice. The level of LDH1 characteristic for damage in cardiac muscle and kidney, LDH2 for kidney, LDH4 for brain and lung, and LDH5 for liver and skeletal muscle were higher in TG2^+/+^ mice-derived samples compared to TG2^-/-^ ones, respectively. When we calculated the relative LDH isoenzyme activity values in serum samples of mice, we found that compared to the other isoenzymes, the elevation of LDH1 level was the most prominent in TG2^+/+^ samples suggesting that the highest difference in organ damages is in the heart. To confirm that the heart was damaged more seriously in TG2^+/+^ animals, we also checked CK-MB activity in sera which is a much more specific marker for heart [90] and found that it was also significantly higher in TG2^+/+^ animals compared to TG2^-/-^ mice. Although our data indicate that the heart was damaged more seriously in TG2^+/+^ animals after phenylephrine treatment, it is important to note that there was no difference in the HR values of the mice, except at a single time point of the experiments in the dark period, which suggest that heart damages did not cause functional disorders in the investigated period. The role of TG2 as a G protein in the maintenance of normal cardiovascular function has not been clarified yet [45]. 

## 4. Materials and Methods

### 4.1. Materials

All chemicals were from Sigma-Aldrich (Munich, Germany) except indicated otherwise.

### 4.2. Mice, Treatments and Obtained Samples

TG2 deficient mice (TG2^-/-^) [44] and WT littermates (TG2^+/+^) with C57BL/6J genetic background were obtained from heterozygous breeding couples and were genotyped in the Animal Core Facility at the University of Debrecen. Mice were housed separately, had ad libitum access to water and chow, and were kept in a 12 h light (6 a.m. to 6 p.m.) and 12 h dark (6 p.m. to 6 a.m.) cycle at 22 ± 1 °C. Body mass of 18-week old males was measured and they were injected intraperitoneally with 60 nM/g body weight phenylephrine (specific alpha1-AR agonist) [91] or CL-316,243 (specific beta-AR agonist) [92]. Mice were anesthetized and blood was collected from the heart, serum was obtained and stored at −80 °C. All the animal experiments were approved by the Animal Care and Use Committee of University of Debrecen (DEMAB) project IDs 14/2010/DEMAB and 1/2014/DEMAB (approved on 18/01/2010 and 26/11/2014, respectively) according to national, and EU ethical guidelines.

### 4.3. Indirect Calorimetry

Measurements were performed using the Comprehensive Lab Animal Monitoring System (CLAMS, Columbus Instruments, Columbus, OH, USA). Six TG2^+/+^ males and six TG2^-/-^ males on a chow diet were placed individually in chambers for 4 consecutive days at ambient temperature (22 °C). Mice were provided free access to food and water, in a 12 h light (6 a.m. to 6 p.m.) and 12 h dark (6 p.m. to 6 a.m.) cycle. Measurements were made in 8 min intervals after the initial 18–20 h acclimation period. Physical activity was monitored in the X planes (XTOT) using an infrared light beam. Measurements for animals’ oxygen consumption (VO_2_) and carbon dioxide production (VCO_2_) were used to estimate the respiratory exchange ratio (RER). Food intake and heat generation were also measured in metabolic chambers [93]. The acclimation period was followed by control measurement for 24 h without treatment. Then, mice were injected intraperitoneally with 60 nM/g body weight phenylephrine (specific alpha1-AR agonist) or CL-316,243 (specific beta-AR agonist) and investigated for an additional 24 h. The average and related standard deviation of obtained values were calculated and statistically analyzed using two-way ANOVA. In the end, all the mice survived the experiment and recovered completely.

### 4.4. Blood Pressure Measurement

The CODA Non-Invasive Blood Pressure system (a tail-cuff method, Kent Scientific Corporation, Torrington, CT, USA) was used to measure the blood pressure (BP) in mice as described [94,95]. The CODA tail-cuff system uses volume pressure recording (VPR) to detect the blood pressure by determining the tail blood pressure (TBP). Special attention is afforded to the length of the occlusion cuff to derive the most accurate blood pressure readings. Three different blood pressure parameters were measured; systolic and diastolic blood pressure and heart rate. Measurements were carried out on awake mice. The acclimation period was followed by control measurements for 24 h without treatment. Then, mice were injected intraperitoneally with 60 nM/g body weight phenylephrine (specific α1-AR agonist) and investigated for an additional 13 h.

### 4.5. Detection of Blood Metabolic Parameters

Blood glucose concentration was measured as previously described [53]. Serum samples were prepared from the plasma of mice collected 13 h after phenylephrine treatment through heart punctuation. Total cholesterol (C), low-density lipoprotein cholesterol (LDL-C), high-density lipoprotein cholesterol (HDL-C), triglyceride (TG), free fatty acid (FFA), and lactate levels were determined by colorimetric enzyme assays (Cobas6000, Roche Ltd., Mannheim, Germany) and free fatty acid (FFA) by a standard laboratory assay [96,97]. Insulin content was detected using Mouse Insulin ELISA Kit (Mercodia, Sweden), glucagon level was measured using Glucagon EIA Kit (Sigma-Aldrich Chemie GMBH, Darmstadt, Germany) according to the manufacturers’ instruction from 5–5 TG2^+/+^ and TG2^-/-^ serum samples.

### 4.6. Detection of LDH and CK-MB

LDH activity was determined with a UV kinetic method recommended by IFCC [98], and CK-MB activity was measured with the immunoinhibition UV kinetic method on the Cobas-501 analyzer (Roche) [99,100]. LDH isoenzymes were separated on Hydragel ISO-LDH with electrophoresis and the amounts of isoenzymes were determined by densitometry (Sebia Hydrasys, Sebia, Lisses, France) [101].

### 4.7. Statistical Analyses

GraphPad Prism version 7.0 and Microsoft Excel 14.0 were used for data interpretation and calculation of significance. Results are expressed as the mean ± SD for the assays indicated. For comparing groups Student’s *t*-test and two-way ANOVA (Tukey’s multiple comparison test) were used. Values of *p* < 0.05 were considered statistically significant with *, ** and *** corresponding to *p* < 0.01 and *p* < 0.001, respectively.

## 5. Conclusions

In this study, we have investigated the pathophysiological effects of the α1-AR agonist phenylephrine in TG2^-/-^ mice and found pieces of evidence that that TG2 participates in α1-AR-regulated biological processes and obtained proofs in vivo that it regulates metabolic and vascular functions. Although our data suggest that TG2 acts as an α1B-AR-coupled G protein, further studies have to reveal details of the underlying molecular mechanisms. 

## Abbreviations

AC	adenylyl cyclase
ACC	acetyl-CoA carboxylase
AMPK	AMP-activated protein kinase
AR	adrenoreceptor
BP	blood pressure
C	total cholesterol
CK-MB	creatine kinase isoenzyme
CL	CL-316,243
CLAMS	Comprehensive Lab Animal Monitoring System
CPT1	carnitine palmitoyltransferase 1
FFA	free fatty acid
GPCR	G-protein-coupled receptor
GTP	guanosine-triphosphate
HDL	high-density lipoprotein
HR	heart rate
IFCC	International Federation of Clinical Chemistry and Laboratory Medicine
KO	knock-out
LDH	lactate dehydrogenase
LDL	low-density lipoprotein
PLCδ1	phospholipase Cδ1
RER	respiratory exchange ratio
TBP	tail blood pressure
TG	triglyceride
TG2	tissue transglutaminase
VCO_2_	carbon dioxide production
VO_2_	oxygen consumption
VPR	volume pressure recording
WT	wild type
XTOT	total X plane cage movements
